# Influence of the N-terminal segment and the PHY-tongue element on light-regulation in bacteriophytochromes

**DOI:** 10.1074/jbc.RA118.007260

**Published:** 2019-01-25

**Authors:** Geoffrey Gourinchas, Uršula Vide, Andreas Winkler

**Affiliations:** From the ‡ Institute of Biochemistry, Graz University of Technology, 8010 Graz, Austria and; § BioTechMed-Graz, 8010 Graz, Austria

**Keywords:** photoreceptor, signal transduction, photobiology, cyclic di-GMP (c-di-GMP), ultraviolet-visible spectroscopy (UV-visible spectroscopy), protein engineering, bilin, diguanylate cyclase, GGDEF, phytochrome

## Abstract

Photoreceptors enable the integration of ambient light stimuli to trigger lifestyle adaptations via modulation of central metabolite levels involved in diverse regulatory processes. Red light–sensing bacteriophytochromes are attractive targets for the development of innovative optogenetic tools because of their natural modularity of coupling with diverse functionalities and the natural availability of the light-absorbing biliverdin chromophore in animal tissues. However, a rational design of such tools is complicated by the poor understanding of molecular mechanisms of light signal transduction over long distances—from the site of photon absorption to the active site of downstream enzymatic effectors. Here we show how swapping structural elements between two bacteriophytochrome homologs provides additional insight into light signal integration and effector regulation, involving a fine-tuned interplay of important structural elements of the sensor, as well as the sensor–effector linker. Facilitated by the availability of structural information of inhibited and activated full-length structures of one of the two homologs (*Idiomarina* species A28L phytochrome-activated diguanylyl cyclase (*Is*PadC)) and characteristic differences in photoresponses of the two homologs, we identify an important cross-talk between the N-terminal segment, containing the covalent attachment site of the chromophore, and the PHY-tongue region. Moreover, we highlight how these elements influence the dynamic range of photoactivation and how activation can be improved to light/dark ratios of ∼800-fold by reducing basal dark-state activities at the same time as increasing conversion in the light state. This will enable future optimization of optogenetic tools aiming at a direct allosteric regulation of enzymatic effectors.

## Introduction

Light is a ubiquitous actuator influencing the development of organisms in all kingdoms of life. Protein-based sensors have evolved to monitor light quality changes in the environment to trigger critical lifestyle adaptations. Light sensing is enabled by a collection of modular photoreceptors responding to the broad spectrum of ambient light (1, 2). These photoreceptors feature a high modularity of output functionalities, and even among members of the same family pronounced differences in photoresponses, chromophore binding, oligomerization, and stability of the photoactivated state are observed (3, 4). Phytochrome sensors, which respond to light in the red and far-red light regime, are involved in the environmental adaptation of plants, algae, fungi, and bacteria (5–7). Because of their properties of reversible photoactivation and deep tissue penetration of red light, phytochromes recently appeared as promising targets for near-IR–based optogenetic applications allowing noninvasive control of biological process *in vivo* (7, 8). The photosensory modules (PSMs)^2^ of bacteriophytochromes are usually composed of a period/ARNT/single-minded (PAS) domain preceded by a N-terminal segment (NTS) that covalently binds the open-chain tetrapyrrole biliverdin chromophore via a thioether linkage with a conserved cysteine residue (9), a cGMP phosphodiesterase/adenylyl cyclase/FhlA (GAF) domain stabilizing the chromophore by polar and hydrophobic interactions, and a phytochrome-specific (PHY) domain stabilizing the photoactivated Pfr state (10, 11). This stabilization is provided by a typical β-hairpin extension, called the PHY-tongue, interacting with conserved residues of the GAF domain (10, 12). Upon illumination, the PHY-tongue element was shown to partially refold to an α-helix following chromophore photoisomerization (10–14). Eventually these structural rearrangements around the chromophore-binding site transduce the molecular signal to a downstream effector module via the PHY domain and its C-terminal sensor–effector linker element (15–17). The initial molecular event leading to phytochrome activation is the photoisomerization of the bilin chromophore around the C15=C16 methine bridge leading to a flip of the D-ring by ∼180° (18). In canonical phytochromes this initial photoisomerization event populates several intermediates along the structural reorganization from the initial dark-state conformation (Pr, chromophore in its *ZZZssa* configuration (19)) to a chromophore and protein environment that represent the light-activated state (Pfr, chromophore in its *ZZEssa* configuration (20)). The property of phytochromes to act as photointerconvertible light switches relies on intricate interactions between the bilin chromophore and the protein environment to eventually transduce light-induced local structural changes to downstream effectors. However, different mechanistic hypotheses have been postulated for long-range signal transduction mechanisms in phytochrome systems, which might be linked to its complexity of involving several structural elements and its evolutionary adaptation to specific light regimes and/or output effectors in various phytochrome species (21–23). The existence of several photocycle intermediates during the photoconversion from Pr to Pfr complicates the interpretation of phytochrome absorption properties with structural changes during light signal transduction. Nevertheless, previous studies on photoconversion of bacteriophytochrome fragments linked the formation of characteristic photocycle intermediates to the reorganization of the chromophore environment (24–27). However, the connection between chromophore isomerization and structural changes in the context of full-length phytochrome dimers still remains poorly understood.

Conserved residues within the chromophore-binding region and at the PHY-tongue–GAF interface have been shown to impact the light response and the stability of the photoactivated state of different phytochrome families (28–31). Recent studies point to an effect of local structural rearrangements around the chromophore influencing the modulation of isomerization routes (32) and photoactivated state stability (33), indicating subtle variations in Pr to Pfr photoconversion within closely related phytochrome species. Beyond the intrinsic properties of phytochrome residues around the chromophore-binding site, their quaternary structure also plays an important role in the modulation of light responses (15, 33–35). Indeed, most phytochromes are dimeric in solution, and frequently the C-terminal effectors and/or the corresponding linker regions are highlighted as important dimer interfaces (15–17). This indicates that an improved understanding of signal transduction mechanisms in full-length phytochromes requires a detailed understanding of the intra- and interprotomer domain interactions controlling stability of the photoactivated state and structural rearrangements in its surrounding. Because of the availability of full-length crystal structures in inhibited and activated conformations (15, 36), the phytochrome-activated diguanylyl cyclase from *Idiomarina* species A28L (*Is*PadC) is a promising target to further address the questions outlined above.

To better appreciate the impact of conserved bacteriophytochrome elements on photoconversion and signal transduction capacities in the full-length context, we exchanged individual domains between two PadC homologs (*Is*PadC and *Ts*PadC from *Thioalkalivibrio* sp. ALMg3) featuring distinctive photoresponses. We highlight the impact of changing interactions between functional PadC domains, surrounding the chromophore-binding site, on photoconversion and signal transduction properties. Notably, we show that the coordination between the NTS and the PHY-tongue elements is, in addition to the intrinsic properties of the GAF domain, a critical factor influencing the spectral diversity of phytochromes. Because of the direct influence of the PHY-tongue conformation on the modulation of the PHY domain and C-terminal linker interfaces (11, 15, 16, 36), we show that also the NTS interaction with the PHY-tongue has important consequences for the light signal transduction in bacteriophytochromes.

## Results

### Characteristic differences in IsPadC and TsPadC photoactivation

**Figure 1.**
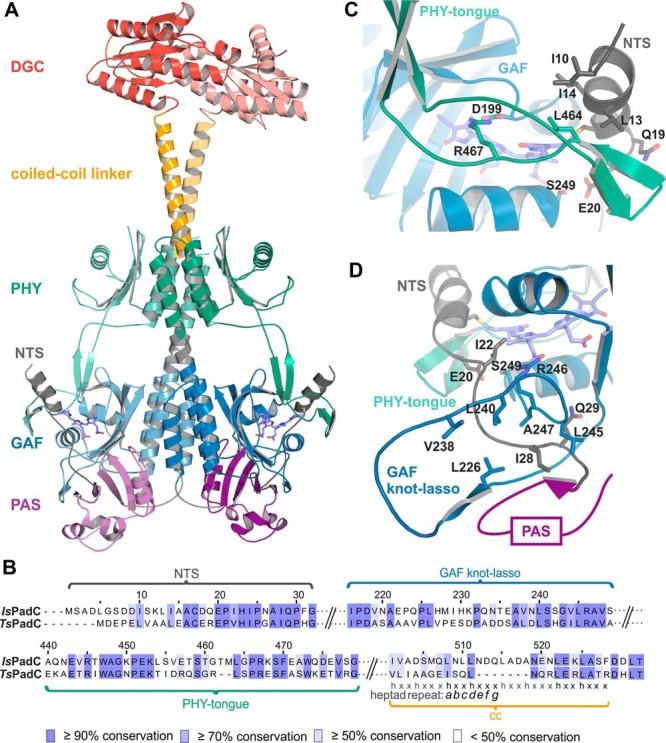
**Conservation of residues between BV surrounding structural elements in *Is*PadC and *Ts*PadC.** *A*, cartoon representation of the dark-adapted full-length structure of *Is*PadC (15). Subdomains are colored as follow: NTS, *black*; PAS, *purple*; GAF, *blue*; PHY, *green*; coiled-coil linker, *orange*; and DGC, *red. B*, sequence alignment generated using Clustal Omega (56) with standard settings and colored by residue conservation using Jalview (57). The alignment is represented for important structural features of the *Is*PadC and *Ts*PadC photosensory modules and the coiled-coil linker regions. *C*, close-up view of the NTS interactions with the GAF domain and the PHY-tongue element in the Pr state of *Is*PadC. *ZZZssa* BV and important residues involved in the interactions are highlighted in stick representation. *D*, close-up view of the typical phytochrome GAF knot lasso of *Is*PadC. Important conserved residues involved in the stabilization of the NTS element are highlighted in stick representation.

**Figure 2.**
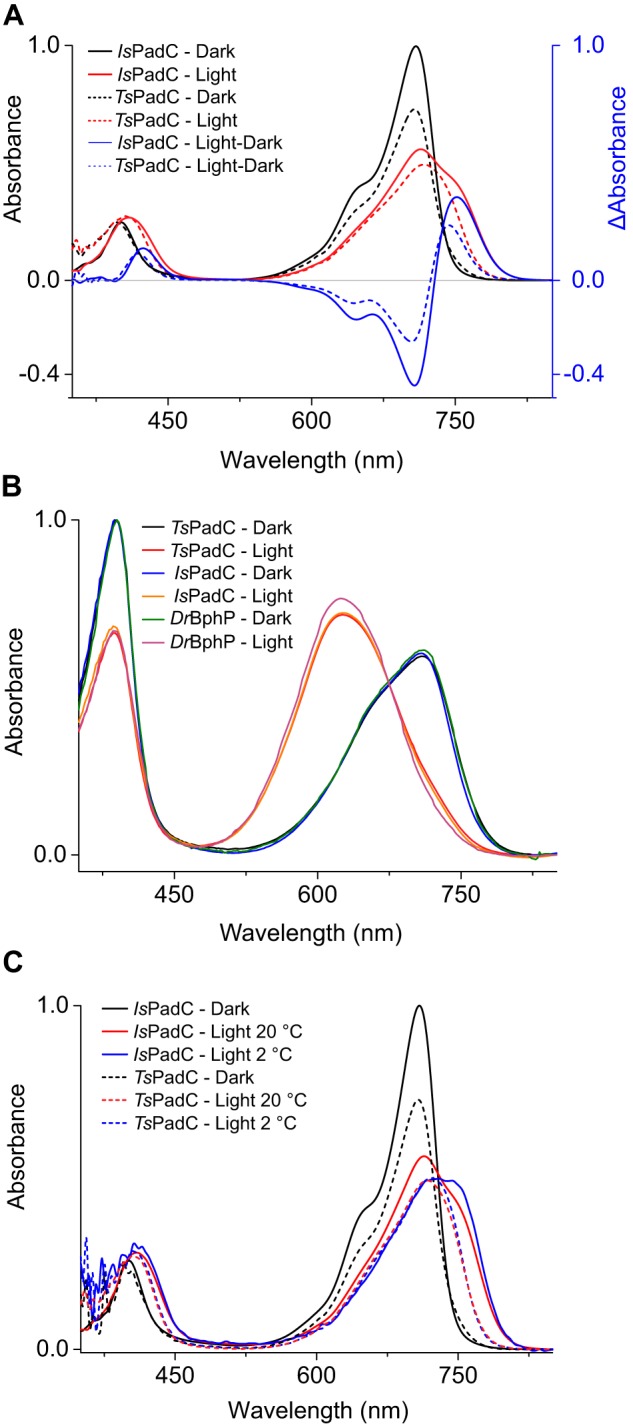
**Comparison of UV-visible absorption spectra of *Is*PadC and *Ts*PadC.** *A*, superposition of *Is*PadC (*solid lines*) and *Ts*PadC (*dashed lines*) UV-visible spectra, normalized based on the *Is*PadC Soret-band maximum. Difference spectra of illuminated and dark-state spectrum are represented in *blue. B*, denaturation in methanol with 0.1% TCA of *Is*PadC and *Ts*PadC under both dark and illuminated conditions. The same denaturation of *Dr*BphP is shown as a reference of complete *ZZEssa* formation upon illumination. *C*, superposition of room temperature (20 °C) and low temperature (2 °C) absorption spectra of *Is*PadC (*solid lines*) and *Ts*PadC (*dashed lines*). Spectra are scaled together based on the Soret peak absorbance.

*Is*PadC and *Ts*PadC share the same domain organization and highly conserved residues around the biliverdin-binding pocket (Fig. 1). Consequently, they are canonical bacteriophytochromes with Pr ground states featuring absorption maxima of the Q-band at 710 and 708 nm, respectively (Fig. 2*A*). However, because of the high stability of the photoactivated state of *Ts*PadC, the dark state spectrum previously reported for this bacteriophytochrome (15) corresponded to a partially activated population and was incorrectly assigned as spectrum of a peculiar ground state of the phytochrome homodimer. However, acidic denaturation of *Ts*PadC revealed a mixture of *ZZZssa* and *ZZEssa* biliverdin isomers, and the slow decrease of the *ZZEssa* population over time under dark conditions allowed us to conclude that *Ts*PadC features a typical Pr-ground state. Similar to photoactivation of *Is*PadC (36), *Ts*PadC also forms a mixture of Pfr and most likely Meta-R–like species that, in contrast to *Is*PadC, is highly stable (Fig. 2*B*, Table 1, and Fig. S1). The difference spectrum of illuminated and dark-state spectra of *Ts*PadC suggested a hypsochromically shifted Pfr state (744 nm) that leads to a distinctive spectral signature of this bacteriophytochrome species (Fig. 2). Interestingly, recording the dark and illuminated spectra of *Ts*PadC at low temperature, to minimize thermal reversion processes, resulted in increased Pfr-like contributions at 744 nm accompanied by a shift of the isosbestic point of the Pr and illuminated photoequilibrium spectra (Fig. 2*C*). This indicates the presence of more than two species contributing to the steady-state illuminated spectra of *Ts*PadC. The same analysis of *Is*PadC at low temperature also showed increased Pfr contribution at 750 nm (Fig. 2*C*).

**Table 1:** **Overview of dark-state recoveries of the constructs used in this study** If not specified differently, all recovery measurements were performed with a protein concentration of 2–3 μm. Because we observed a suboptimal fit of the dark-state recoveries using the sum of two exponentials for fitting the recoveries of some constructs, we fit the dark-state recoveries as the sum of three exponentials where needed and obtained reproducible amplitudes and time constants for all three contributing processes. After red light illumination of 1 min, changes in absorbance of the maximum of the Pr-state Q-band were followed over at least 60 min, with automatic sampling every 5 s and an integration time of 0.01 s. The contribution of each phase in the thermal recovery is represented as relative amplitude. Because of the influence of the measuring light especially on slow recovering constructs and experimental limitations for fitting very fast recovering constructs, the individual time constants should only be compared in a qualitative manner (refer to “Experimental procedures”). Similarly, the error indicators merely reflect the quality of the fit for a single experiment. The S.E. of the estimate from the nonlinear curve fit corresponding to *y* = *A*_1_*exp(−*x*/τ_1_) + *A*_2_*exp(−*x*/τ_2_) + *A*_3_*exp(−*x*/τ_3_) + *y*_0_ was used as error indicator. Representative raw data and the corresponding fits of thermal recoveries for selected constructs can be seen in Fig. S1.

Constructs	Dark-state recovery measured at maximum Pr Q-band absorption					
	τ_1_	Relative A_1_	τ_2_	Relative A_2_	τ_3_	Relative A_3_
	*s*	%	*s*	%	*s*	%
*Is*PadC (36)	18.5 ± 0.4	34 ± 0.5	100.0 ± 0.7	66 ± 0.5		
*Is*PadC^+7KE^	19.5 ± 0.1	61.7 ± 0.4	81.5 ± 0.6	16.4 ± 0.6	2,200 ± 5	21.9 ± 0.1
*Is*^N/PG/Yt/Y/CC^*Ts*^D^	31.2 ± 0.2	67.6 ± 0.5	207 ± 3	15.5 ± 0.4	5,400 ± 30	16.8 ± 0.1
*Is*^N/PG/Yt/Y/CCΔ515–521^*Ts*^D^	17.5 ± 0.2	36.2 ± 0.5	75.4 ± 0.3	58.3 ± 0.5	1,300 ± 10	5.5 ± 0.1
*Is*^N/PG/Yt/Y/CC501–507^*Ts*^CC505–518/D^	21.5 ± 0.1	45.8 ± 0.4	112 ± 1	22.4 ± 0.4	2,800 ± 10	31.7 ± 0.1
*Is*^N/PG/Yt/Y^*Ts*^CC/D^	15.2 ± 0.2	40.9 ± 0.7	350 ± 1	48.3 ± 0.2	6,800 ± 100	10.8 ± 0.1
*Is*^N/PG^*Ts*^Yt/Y/CC/D^	16.9 ± 0.2	38.9 ± 0.7	420 ± 2	48.6 ± 0.2	110,000 ± 30,000	12.5 ± 0.1
*Ts*^N^*Is*^PG/Yt/Y/CC^*Ts*^D^	418 ± 2	28.5 ± 0.3	2,470 ± 10	55.3 ± 0.3	14,300 ± 100	16.2 ± 0.3
*Ts*^N^*Is*^PG^*Ts*^Yt/Y/CC/D^	19 ± 2	39.0 ± 0.5	108 ± 3	61.0 ± 0.5		
*Ts*^N^*Is*^PG^*Ts*^Yt^*Is*^Y/CC/D^	6.9 ± 0.1	92.9 ± 0.6	56 ± 2	7.1 ± 0.4		
*Ts*^N/PG/Yt/Y^*Is*^CC/D^	576 ± 7	17.4 ± 0.5	1,900 ± 4	82.6 ± 0.5		
*Ts*^N/PG/Yt/Y/CC^*Is*^D^	30.2 ± 0.1	17.5 ± 0.4	1,800 ± 50	11.8 ± 0.5	14,200 ± 300	70.7 ± 0.5
*Ts*PadC	33.4 ± 0.1	27.4 ± 0.4	1,600 ± 20	7.7 ± 0.2	63,000 ± 500	64.9 ± 0.3
*Is*PadC^PSM^	19.4 ± 0.2	35.7 ± 0.2	950 ± 30	12.1 ± 0.4	6,300 ± 90	52.1 ± 0.4

Strikingly, the denaturation of both illuminated bacteriophytochromes under acidic conditions leads to more than 90% of the biliverdin chromophore in *ZZEssa* configuration, comparable with the symmetrically activated *Deinococcus radiodurans* bacteriophytochrome (*Dr*BphP) featuring a classical Pfr spectrum of both protomers (11, 13) (Fig. 2*B*). These observations support that both *Is*PadC and *Ts*PadC feature heterogeneous chromophore environments, even though both biliverdin species of the activated PadC dimer are isomerized. However, molecular mechanisms involved in stabilizing the asymmetric dimer conformation differ for *Is*PadC and *Ts*PadC and result in striking differences in stability of the photoactivated states. Indeed, *Is*PadC recovers to its ground state on a minute time scale, whereas *Ts*PadC requires several hours (Table 1). Because the direct coordination shell of the biliverdin cofactor is almost identical, we anticipated that other structural determinants could account for these characteristic differences.

### The design of PadC chimeras

**Figure 3.**
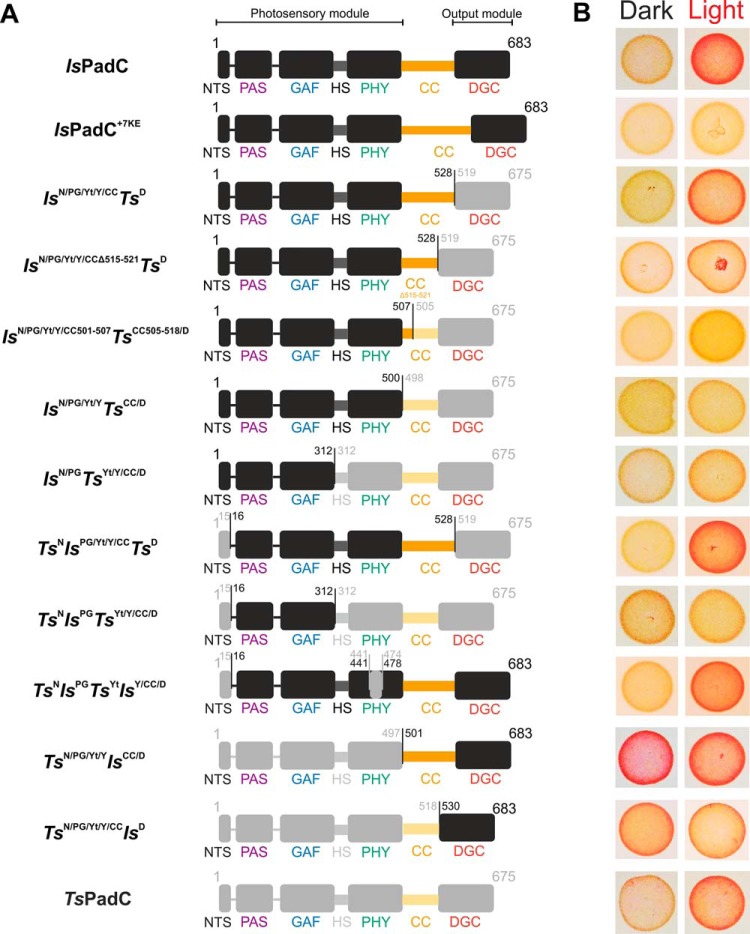
**Overview of generated constructs.** *A*, schematic representation of the domain arrangement of naturally occurring constructs and different synthetic chimeras. Subdomain representations of *Is*PadC and *Ts*PadC are colored in *black* and *light gray*, respectively. The coiled-coil regions are colored in *orange* and *light orange* for *Is*PadC and *Ts*PadC, respectively. Residue numbers of the fusion points are indicated. Names of individual subdomains are colored according to Fig. 1*A. B*, *in vivo* screening of DGC activity in Congo Red–based assays under dark, as well as constant red light illumination. Bacterial colonies expressing active DGCs are colored *red* because of the Congo Red dye being complexed by exopolysaccharides that are produced upon c-di-GMP formation (58). Isolated bacterial colonies are shown in the order of the construct presented in *A*. The full screening plates with all colonies together are available in Fig. S2.

For unambiguous assignment of individual *IsTs* chimeras, a nomenclature is used throughout the manuscript that indicates structural elements in superscript as N, PG, Yt, Y, CC, and D for the NTS, the PAS–GAF core, the PHY-tongue, the PHY domain, the coiled-coil linker, and the diguanylyl cyclase (DGC), respectively. Specific residue numbers of the fusion points are indicated in Fig. 3*A*. The bacteriophytochrome species from which the domains originate precede the domain symbols and are represented as *Is* or *Ts* for *Is*PadC and *Ts*PadC, respectively. Specific residue deletions or additions are specified by residue range numbers (Fig. 3*A*). The effect of the different fusions on red light modulation of DGC activity was screened *in vivo* in *Escherichia coli* and is shown in Fig. 3*B* and Fig. S2.

### The impact of the output domain as well as the coiled-coil linker composition

Because of the primordial role of the coiled-coil linker in the structural asymmetry observed for *Is*PadC (36), we first addressed the effect of effector and coiled-coil linker swapping on the photoactivated state stability. The exchange of DGC effectors between *Is*PadC and *Ts*PadC starting right from the conserved D*X*LT motif of the GGDEF domains (*Is*^N/PG/Yt/Y/CC^*Ts*^D^ and *Ts*^N/PG/Yt/Y/CC^*Is*^D^) pronouncedly affected the stability of the photoactivated state. In fact, light stimulation of DGC activity was barely affected by the exchange of effectors; however, the thermal relaxation to the Pr ground state showed pronounced changes. This indicates that molecular interactions at the DGC dimer interface contribute to the stability of the photoactivated state. This was further supported by the DGC effector truncation of *Is*PadC (*Is*PadC^PSM^) that showed a slower thermal recovery compared with the full-length WT construct (Table 1).

Interestingly, previously generated coiled-coil truncated variants of *Is*PadC (15) showed a release of dark state inhibition of DGC activity, suggesting that the coiled-coil dimer interface has been evolutionary adapted to provide efficient effector inhibition in dark and activation upon coiled-coil register switching under red light illumination. Central coiled-coil residues involved in this mechanism are also conserved in *Ts*PadC (Fig. S3). In fact, the previously described *Is*PadC^Δ514–520^ construct (15) features a truncation of seven residues in its coiled-coil linker region, resulting in the same linker length as *Ts*PadC, and shows an increase in dark-state activity correlating with a reduced stability of the inhibiting register of the coiled-coil linker. Interestingly, the construct *Is*^N/PG/Y/Yt/CCΔ515–521^*Ts*^D^ of this study, which features the same linker length as *Is*PadC^Δ514–520^but is coupled to the *Ts*PadC DGC effector, showed strongly inhibited DGC activity under dark conditions. Considering the fact that *Is*PadC^Δ514–520^ and *Is*^N/PG/Y/Yt/CCΔ515–521^*Ts*^D^ feature almost identical coiled-coil linker registers, with the only exception of an Asn^514^-to-Gln substitution, their differences in dark-state inhibition capacity can only originate from the coevolution of the coiled-coil linker composition with specific DGC properties that influence effector dimerization and consequently the catalytic mechanism at its relatively open dimer interface (37–39).

Along the same line, the construct *Is*PadC^+7KE^ revealed that increasing the stability of the inhibiting register by introducing an additional heptad unit together with salt bridges between the two coiled-coil helices allowed an even stronger inhibition of the naturally coupled DGC effector (Table 2). The introduction of salt bridges between Glu^514^ and Lys^519^ of the respective other protomer (between heptad residues *e* and *g*′ of the inhibiting register sequence, respectively) showed that the switching between inhibiting and stimulating coiled-coil register is crucial to enable DGC activation in PadCs. Interestingly, the reduced conformational flexibility of the coiled-coil also results in a slowed-down thermal recovery of this construct (Table 1). All these observations highlight the fact that the coiled-coil linker composition is an important element for providing a high dynamic range of DGC activation and for controlling overall output activities.

**Table 2:** **Comparison of natural PadCs and synthetic fusions with respect to kinetics of substrate conversion**

Constructs	Initial rates at 200 μm GTP*^a^*		Fold activation
	Dark	Light	
	*(*μ*mol product) min*^−*1*^ *(*μ*mol enzyme_2_)*^−*1*^		
*Is*PadC	1.7 ± 0.2	32 ± 3	19-fold
*Is*PadC^+7KE^	0.13 ± 0.04	0.08 ± 0.01	
*Is*^N/PG/Yt/Y/CC^*Ts*^D^	0.55 ± 0.04	9.0 ± 0.2	16-fold
*Is*^N/PG/Yt/Y/CCΔ515–521^*Ts*^D^	0.02 ± 0.01	17.0 ± 0.1	800-fold
*Is*^N/PG/Yt/Y/CC501–507^*Ts*^CC505–518/D^	0.02 ± 0.01	4.6 ± 0.8	200-fold
*Is*^N/PG/Yt/Y^*Ts*^CC/D^	0.25 ± 0.02	1.09 ± 0.07	4-fold
*Is*^N/PG*Ts*Yt/Y/CC/D^	1.9 ± 0.5	8.11 ± 0.01	4-fold
*Ts*^N^*Is*^PG/Yt/Y/CC^*Ts*^D^	1.04 ± 0.03	17.2 ± 0.6	16-fold
*Ts*^N^*Is*^PG^*Ts*^Yt/Y/CC/D^	0.6 ± 0.04	1.0 ± 0.1	2-fold
*Ts*^N^*Is*^PG^*Ts*^Yt^*Is*^Y/CC/D^	0.07 ± 0.01	55 ± 1	800-fold
*Ts*^N/PG/Yt/Y*Is*CC/D^*^b^*	10.9 ± 0.1	9 ± 1	
*Ts*^N/PG/Yt/Y/CC^*Is*^D^	15 ± 1	34 ± 1	2-fold
*Ts*PadC	8.0 ± 0.7	52 ± 3	7-fold

*^a^* Comparison of product formation between the various constructs was performed for initial reaction rates at 200 μm GTP and after normalization to the dimeric protein concentration. Initial rates are quantified from experimental triplicates for three time points, and the sample standard deviation of individual points contributed to the error estimation of the linear fit that is used to calculate the initial rate of product formation. The S.E. of the estimate from the linear regression is used as an error indicator.

*^b^* Dark-state kinetics of this construct have been measured from the monomer fraction featuring only the Pr state.

**Figure 4.**
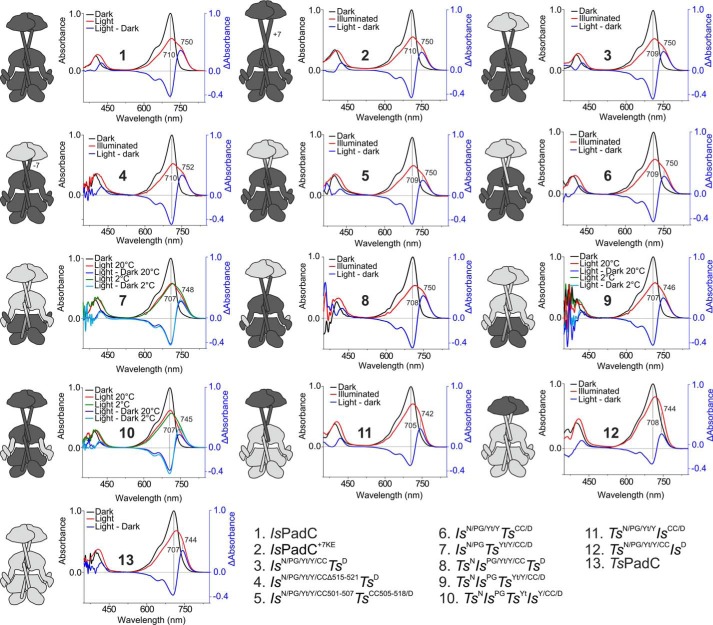
**UV-visible absorption spectra of *Is*PadC and *Ts*PadC as well as synthetic chimeras.** The dark-state spectrum is colored *black*, whereas the illuminated steady-state spectrum is colored *red*. The difference spectrum between the illuminated and dark states is colored *blue*, and the maxima of positive and negative differences are indicated. On the *left side* of each spectrum, a schematic representation of the synthetic fusion is shown where *black* domains derive from *Is*PadC, and *light gray* domains derive from *Ts*PadC. For the fusions *Is*^N/PG^*Ts*^Yt/Y/CC/D^, *Ts*^N^*Is*^PG^*Ts*^Yt/Y/CC/D^, and *Ts*^N^*Is*^PG^*Ts*^Yt^*Is*^Y/CC/D^, UV-visible spectra of the illuminated state in *green* were also recorded at 2 °C to minimize the effect of spontaneous thermal reversion of Pfr states and to reduce shunt reaction pathways from photocycle intermediates. The difference spectrum between the illuminated and dark state at 2 °C is colored *light blue*.

Following the same rationale, the coiled-coil linker composition is equally important for the interplay with the PHY dimer interface and for integrating the incoming light signal of the phytochrome sensor. Indeed, exchanging the coiled-coil linkers together with the DGC effectors (*Is*^N/PG/Yt/Y^*Ts*^CC/D^ and *Ts*^N/PG/Yt/Y^*Is*^CC/D^) resulted in photoactivation properties and absolute enzymatic activities that were strongly decreased in both chimeras (Table 2). Most probably the exchange of the coiled-coil linker region interfered with the correct establishment of the PHY dimer interface, preventing the postulated translation mechanism of the coiled-coil linkers (36). This hypothesis is supported by a variant of *Is*^N/PG/Yt/Y^*Ts*^CC/D^, for which the first *Ts*PadC heptad repeat was replaced by the corresponding heptad repeat of the *Is*PadC linker (*Is*^N/PG/Yt/Y/CC500–507^*Ts*^CC505–675/D^). Indeed, this variant showed a re-establishment of DGC photoactivation capacity similar to *Is*^N/PG/Yt/Y/CC^*Ts*^D^, even though no change in linker length or expected stability of inhibiting and stimulating registers has been introduced. A proper coordination of the PHY dimer interface with the coiled-coil linker is, in fact, also crucial for the stabilization of the dimer interface of the full-length protein as observed for the variant *Ts*^N/PG/Yt/Y^*Is*^CC/D^ where the replacement of the coiled-coil linker region impaired the dimerization of the full-length protein in its dark state conformation (Fig. S4). Interestingly, illumination of this construct allowed the formation of a more stable dimer interface, further supporting the fact that the properties of the dimer correlate with the photoactivation state of the biliverdin environment and, thereby, are critical for regulating DGC activity as well as signal integration by the phytochrome sensor (Fig. 4 and Fig. S4). Unexpectedly, this specific construct, however, shows no significant differences between dark- and light-state activities (Table 2). The relatively high basal activity even in the predominantly monomeric dark-state population suggests that some transient dimer formation occurs even in the absence of light. Because red light–induced dimerization does not directly influence the specific activity, the observed uncoupling further highlights the complex interplay of several structural elements required for signal integration and effector regulation in PadCs (Table 2).

In the light of our previous analysis of the *Is*PadC dimer interface (15, 36), the coiled-coil linker is one driving force for dimerization of the full-length protein. The transition of coiled-coil dimer conformations is directly coupled to the conformational state of the PHY-tongue element as observed from the structures of *Is*PadC and the *Is*PadC^Reg2^ variant (15, 36). Therefore we also looked in more detail at the involvement of the PHY-tongue elements and the PHY dimer interface.

### A direct interaction of the PHY-tongue and the NTS in bacteriophytochromes

Different PHY-tongue conformations have been linked to the isomerization state of the biliverdin chromophore (10, 13, 29). However, considering the broad diversity of photoactivated state stabilities in various phytochromes, it is obvious that multiple interactions are involved in stabilizing the various conformations and that addressing the interactions between the PHY-tongue and its surrounding GAF and NTS modules is one way to better understand the interplay of these central phytochrome elements in light signal transduction.

**Figure 5.**
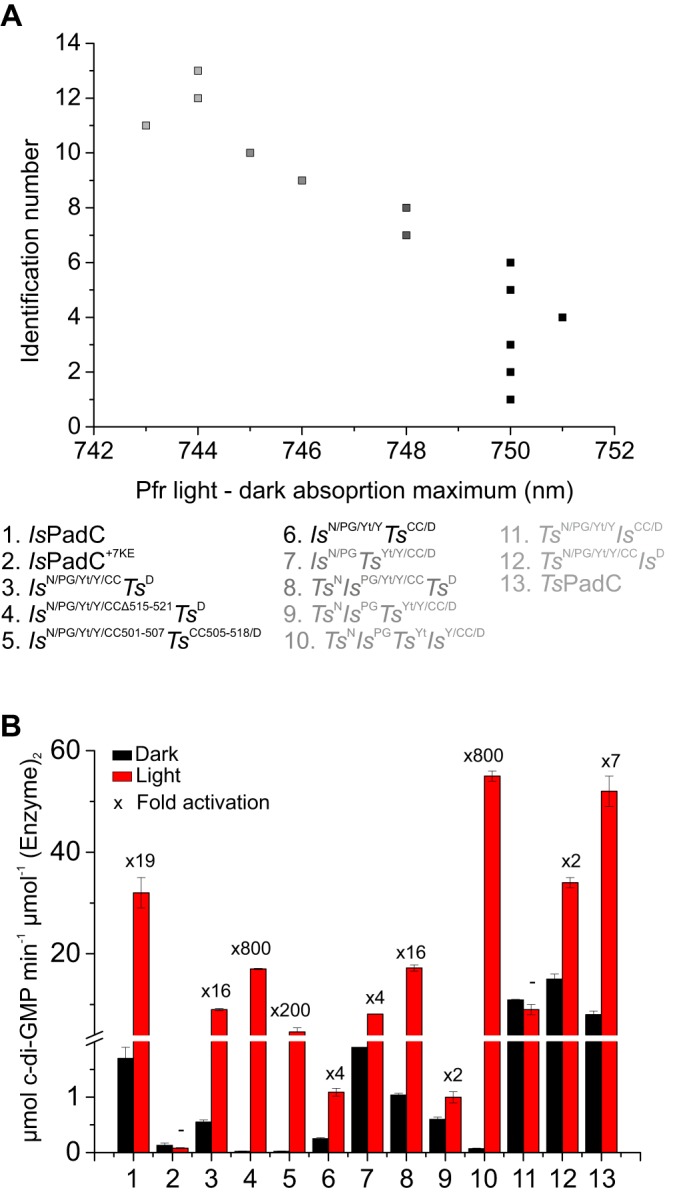
**Regulation capacity of DGC activity.** *A*, maxima of the far-red light absorption derived from the difference between illuminated and dark state spectra of all constructs. *B*, comparison of apparent turnover rate constants between dark and light conditions at 200 μm GTP. The fold activation is indicated *above* the columns. Initial rates are quantified from experimental triplicates of three time points, and the *sample* standard deviation of individual points contributed to the error estimation of the linear fit that is used to calculate the initial rate of product formation. The S.E. of the estimate from the linear regression is used as an error indicator.

Therefore, we generated a series of chimeras of which the variant *Is*^N/PG^*Ts*^Yt/Y/CC/D^ features the chromophore-binding core of *Is*PadC in the context of the PHY, CC, and DGC domains of *Ts*PadC. In this construct, the PHY-tongue of *Ts*PadC interacts with the chromophore-binding core of *Is*PadC. Although the spectral properties of this variant appeared at a first glance rather similar to *Is*PadC WT, the variant showed a hypsochromic shift of the Q-band absorption toward that of the *Ts*PadC WT (Fig. 4 and Fig. 5*A*). Moreover, the variant did not show a pronounced effect of low temperature measurement on the increase of chromophore environment homogeneity as compared with *Is*PadC, suggesting different stabilization of the chromophore environments in this variant (Fig. 4).

The adaptation of the PHY-tongue to its proximal environment is also highlighted by the fact that the variant *Is*^N/PG^*Ts*^Yt/Y/CC/D^ was relatively poorly expressed in *E. coli* compared with the *Is*PadC and *Ts*PadC wildtypes. Interestingly, the additional introduction of the *Ts*PadC NTS in the *Is*^N/PG^*Ts*^Yt/Y/CC/D^ variant generated the construct *Ts*^N^*Is*^PG^*Ts*^Yt/Y/CC/D^, which enables comparable expression yields to the *Ts*PadC WT. Because we had previously observed that the apo-form of full-length *Is*PadC is unstable during expression in *E. coli*, the differences in expression between these two variants could indicate that an appropriate interaction between the PHY-tongue and the NTS elements is critical for the efficient loading of the chromophore to its GAF-binding pocket. Strikingly, *Ts*^N^*Is*^PG^*Ts*^Yt/Y/CC/D^ had its Q-band absorption even further shifted toward the *Ts*PadC WT properties, which indicates a contribution of the NTS conformation on the absorption properties of the cofactor by modulating its environment (Figs. 4 and 5*A*). Moreover, the variant *Ts*^N^*Is*^PG^*Ts*^Yt/Y/CC/D^ showed a much faster thermal recovery than *Is*^N/PG^*Ts*^Yt/Y/CC/D^, further supporting the interplay of NTS, PHY-tongue, and cofactor environment and their coordinated influence on photocycle properties.

These conclusions are also supported by the variant *Ts*^N^*Is*^PG/Yt/Y/CC^*Ts*^D^, which features the *Ts*PadC NTS in the context of the *Is*PadC PSM. This variant showed an identical hypsochromic shift of the Pr-state Q-band absorption as observed for *Ts*PadC but an illuminated spectrum resembling the *Is*PadC WT (Figs. 4 and 5*A*). The thermal recovery, however, was slowed down significantly compared with *Is*PadC or *Is*^*N*/PG/Yt/Y/CC^*Ts*^D^ (Table 1). Interestingly, the exchange of only the NTS and PHY-tongue elements in *Is*PadC (*Ts*^N^*Is*^PG^*Ts*^Yt^*Is*^Y/CC/D^) generated similar *Ts*PadC-like spectral properties as observed for *Ts*^N^*Is*^PG^*Ts*^Yt/Y/CC/D^, further supporting the importance of the conformational adaptation of NTS and PHY-tongue elements for light signal integration. At the same time, the photoactivated state of this variant is rather short-lived, indicating that the α-helical conformation of the PHY-tongue is destabilized in this variant (Table 1 and Fig. S1). Because the GAF and NTS domains are identical for *Ts*^N^*Is*^PG^*Ts*^Yt^*Is*^Y/CC/D^ and *Ts*^N^*Is*^PG^*Ts*^Yt/Y/CC/D^, this destabilization might originate from an uncoupling of the PHY-tongue element with the PHY domains, highlighting the importance of the PHY-dimer interface and its coupling with the PHY-tongue conformation.

### The PAS–GAF core as central element in photoactivation

All the generated constructs so far indicated an influence of the PHY-tongue and the NTS on the absorption properties of the full-length protein. However, essential structural information influencing the absorption properties of phytochromes is encoded in the local environment of the biliverdin at the GAF domain. Nevertheless, both *Ts*^N^*Is*^PG^*Ts*^Yt/Y/CC/D^ and *Ts^N^Is^PG^Ts^Yt^Is^Y^*^/CC/D^ that include the *Is*PadC GAF domain featured spectral signatures with indications of *Ts*PadC-like characteristics but still quite different thermal reversion kinetics. The analysis of amino acid conservation between *Is*PadC and *Ts*PadC showed that all critical residues interacting with the biliverdin chromophore and stabilizing its ZZ*Zssa* conformation are conserved (15). Based on the activated protomer structure of *Is*PadC^Reg2^ (36), important residues not directly interacting with the ZZ*Zssa* biliverdin but changing rotamer upon ZZ*Essa* chromophore isomerization appear to be conserved as well. Only residues Met^258^ and Tyr^189^ in *Ts*PadC (replaced by the Phe^259^ and Met^190^ in *Is*PadC, respectively) are notable residue changes potentially affecting the biliverdin D-ring.

### Kinetics of c-di-GMP formation

All but two constructs show red light activation of c-di-GMP formation (Fig. 5*B*). Some constructs, such as *Is*^N/PG^*Ts*^Yt/Y/CC/D^,*Ts*^N^*Is*^PG^*Ts*^Yt/Y/CC/D^, *Ts*^N/PG/Yt/Y^*Is*^CC/D^, and *Ts*^N/PG/Yt/Y/CC^*Is*^D^, feature a high DGC activity already in the dark with little difference to the light state activity, suggesting a reduced stabilization of the inhibited PadC dimer. Other constructs, such as *Is*^N/PG/Yt/Y/CCΔ515–521^*Ts*^D^, *Is*^N/PG/Yt/Y/CC501–507^*Ts*^CC505–518/D^, and *Ts*^N^*Is*^PG^*Ts*^Yt^*Is*^Y/CC/D^, show a stronger inhibition of DGC activity in the dark state. Although *Is*^N/PG/Yt/Y/CCΔ515–521^*Ts*^D^ and *Is*^N/PG/Yt/Y/CC501–507^*Ts*^CC505–518/D^ also have a lower light state DGC activity, *Ts*^N^*Is*^PG^*Ts*^Yt^*Is*^Y/CC/D^ revealed that high DGC activation under illumination is compatible with a strong inhibition in dark.

Because an 800-fold difference of DGC activity has been observed for this construct, where only the NTS and the PHY-tongue element have been modified, it appears as if the balance between inhibited and activated conformations of the catalytic DGC dimer is influenced by a complex interplay of structural elements communicating with regions proximal to the biliverdin-binding site. A similar dynamic range is also observed for construct *Is*^N/PG/Yt/Y/CCΔ515–521^*Ts*^D^, where the coiled-coil sensor effector linker is shortened by one heptad unit. This again highlights the importance of the sensor–effector linker in addition to the structural elements close to the biliverdin-binding site.

Although it is difficult to infer common rules from the different chimeras, it is clear that subtle differences between constructs can have pronounced effects on effector regulation and/or spectral properties. In line with our observations, the residue composition of the GAF fold is primordial for the positioning of the pyrrole rings in the chromophore-binding pocket; however, spectral properties and photoactivated state stabilities of full-length bacteriophytochromes appear to be strongly influenced by structural elements in the vicinity of the chromophore-binding site. Notably, the NTS and the PHY-tongue element interact with each other and thereby appear to be crucial elements in the stabilization of Pr and photoactivated states of bacteriophytochromes.

## Discussion

Considering PadCs as powerful model systems for phytochrome function, where structural studies have recently improved the understanding of molecular details involved in the long-range signal transduction mechanisms of phytochromes (15, 36), we addressed the involvement and cross-talk between characteristic structural elements in bacteriophytochrome signaling, the NTS, and the PHY-tongue. Our findings highlight both elements as critical regions for tuning photoresponses in full-length phytochrome systems with otherwise highly conserved chromophore-binding regions in the GAF domain.

The central role of the GAF domain had previously been demonstrated by the replacement of two residues within the GAF domain of *Agrobacterium tumefaciens* bathy-phytochrome Agp2 resulting in a canonical character (40). Similarly, the substitution of Gln^188^ by a Leu residue in the GAF domain of the *Pseudomonas aeruginosa* bathyphytochrome strongly impaired the stability of the Pfr state under dark conditions, reducing the bathy character of this bacteriophytochrome variant (10). The dominance of the GAF domain in defining bacteriophytochrome photoresponses is also reflected in our synthetic fusions because their spectral signatures appear similar to the absorption characteristics of the WT bacteriophytochrome from which the GAF domain originates. In fact, the characteristics of heterogeneous chromophore environments in the light-activated state, which is reflected in the typical photoequilibria of our dimeric phytochrome species, is conserved in all of our synthetic fusions. Therefore, the amino acid composition of the PAS–GAF core might be one of the key players in the asymmetric behavior of certain bacteriophytochrome species (36). Strikingly, low-temperature UV-visible spectra of both WT constructs showed an increased Pfr contribution and thus a decrease of heterogeneity in the chromophore environment, which supports allosteric communication between the two protomers enabling structural asymmetry in dimeric phytochromes. Moreover, the different domain assemblies, provided by the various PadC designs described here, feature different spectral responses to low temperature measurements. For instance, the variant *Ts*^N^*Is*^PG^*Ts*^Yt/Y/CC/D^ features similar dark-reversion properties as *Is*PadC WT but does not show an increased Pfr contribution at low temperature, indicating that changes at the dimer interface influence the allosteric communication between the phytochrome protomers.

Strikingly, we also observed that the modulation of the PHY-tongue interactions with the GAF domain, as well as with the NTS generated interesting variations in the absorption properties of chimeric bacteriophytochromes. This suggests an important regulatory involvement of the structural elements positioned near the biliverdin-binding pocket.

### The NTS and the PHY-tongue element influence Pr- and Pfr-state properties

Our synthetic fusions *Ts*^N^*Is*^PG^*Ts*^Yt/Y/CC/D^ and *Ts*^N^*Is*^PG^*Ts*^Yt^*Is*^Y/CC/D^ showed that the NTS and the PHY-tongue elements have a strong influence on the absorption properties of the full-length holoprotein. Notably, the fact that the construct *Ts*^N^*Is*^PG/Yt/Y/CC/D^, featuring the *Ts*PadC NTS only, showed a subtle hypsochromic shift in spectral signature indicative of *Ts*PadC and that the construct *Is*^N/PG^*Ts*^Yt/Y/CC/D^ and *Ts*^N^*Is*^PG^*Ts*^Yt^*Is*^Y/CC/D^, both featuring the *Ts*PadC PHY-tongue, in addition showed similarity with the *Ts*PadC spectral signature supported the important role of the PHY-tongue element in modulating absorption properties of full-length bacteriophytochromes. Nevertheless, the combination of the NTS and PHY-tongue elements from the same species allows a stronger hypsochromic shift of the absorption properties toward a *Ts*PadC-like spectral signature (*cf. Ts*^N^*Is*^PG^*Ts*^Yt/Y/CC/D^ and *Ts*^N^*Is*^PG^*Ts*^Yt^*Is*^Y/CC/D^), suggesting an important molecular coordination between the NTS and the PHY-tongue during photoactivation. A complex interplay between the GAF domain and the PHY-tongue is required to transition from the Pr-ground state to the final Pfr photoproduct through various intermediate states (25, 26). It appears from our bacteriophytochrome fusions that the NTS also has an important role in this interplay. Interestingly, apart from the NTS region of the *D. radiodurans* bacteriophytochrome, which does not show characteristic conformational changes upon protomer activation (11, 13), other phytochromes species feature conformational changes upon photoactivation (36, 41) that resemble the NTS conformation of bathyphytochromes (10, 42, 43). Therefore, the N-terminal segment is assumed to have a profound impact on Pr- and Pfr-state stabilities in bacteriophytochromes.

It has also been shown for plant phytochromes that the deletion or the modification of the N-terminal extension (NTE), corresponding to longer versions of the NTS region in bacteriophytochromes, has profound effects on the Q-band absorption and thermal recovery properties (44–47). Recent structures of Pr and Pfr states of a plant phytochrome also support a change in NTE conformation upon refolding of the PHY-tongue, supporting the important molecular coordination of NTE and PHY-tongue also in plant phytochromes (41). Moreover, mutational studies of *Arabidopsis thaliana* PhyB revealed that the mutation of residues located at the beginning of the PAS domain and in narrow interaction with the GAF knot-lasso severely impaired the light-induced PIF3 binding capacity of full-length PhyB (48). Because the interaction between the GAF knot-lasso and the PAS N terminus might be important for the correct positioning of the NTE element, the plant NTE might also play an important role in the biological function of phytochromes.

Because of these emerging universal effects of the N-terminal regions on phytochrome properties in the Pr state, as well as in the Pfr to Pr thermal recovery rate, the N-terminal elements and the previously described central regulatory PHY-tongue element appear as characteristic structural elements that can be altered to tune phytochrome properties. Among others, interesting targets would be the reduction of spectral overlap between Pr and Pfr states or the modulation of dynamic ranges or basal activities in full-length phytochrome-coupled enzymatic systems, without interfering with functionally critical residues located near the biliverdin-binding site.

### DGC regulation by phytochrome sensors

From our synthetic constructs it appeared that individual GGDEF domains can be exchanged between various PadC homologs without disturbing the dynamic range of light activation and retaining basal activities characteristic for each system. At the same time, the coiled-coil sensor–effector linker was highlighted as a central structural element required for the modulation of enzymatic activity by light. Notably, the composition of the linker in addition to its length seemed to have a profound impact on the efficiency of signal transduction, as well as on defining the level of dark state enzymatic activity. With respect to the previously proposed mechanism of coiled-coil register switching upon PSM activation (36), the composition of the coiled-coil can affect the stability of either the inhibiting or stimulating register and is therefore directly reflected in the dynamic range of DGC activation. Interestingly, *Is*PadC and *Ts*PadC both have conserved residues in critical coiled-coil positions, even though the *Ts*PadC linker is shorter by one heptad unit. Therefore, a similar equilibrium between inhibiting and stimulating coiled-coil registers is expected for *Ts*PadC, as previously described for *Is*PadC (15) (Fig. S3). Because similar helical linker register transitions have been described for other photoreceptors (35), the amino acid composition of the linker element is an important but difficult to rationalize feature that requires special attention in the design of synthetic fusions of photoreceptors with different effector outputs. Moreover, the description of rotational (16, 49), translational (17, 36), or unfolding (50) transitions of the helical linker element suggest that there might not be a specific mode of enzyme regulation by phytochromes. Eventually, these aspects challenge the fusion of non-natural phytochrome sensors with enzymatic effectors; however, the observed natural modularity of sensor–effector couples suggests that suitable linker elements for enabling effective signal transduction can be obtained for diverse systems.

As far as the signal integration by the helical linker is concerned, the proper coupling of the PHY-tongue element with the dimeric interface of the PHY domains also appears to be crucial. As observed for *Is*^N/PG/Yt/Y^*Ts*^CC/D^ and *Ts*^N/PG/Yt/Y^*Is*^CC/D^, both fusions showed a strongly reduced DGC activity, although the respective *Is*PadC and *Ts*PadC coiled-coil linker sequences are expected to operate via similar linker translation mechanisms. This highlights the importance of the PHY dimer interface and its compatibility with structural changes originating from the biliverdin environment, as well as its communication with the sensor–effector linker in light signal transduction. Because the PHY dimer interface is influenced by the positioning of the central helical spine, connecting the GAF domains with the PHY domains, the conformational dynamics of the helical spine also influence the signal transduction mechanism. In this respect, the low evolutionary conservation of the central helical spine and the dimerization interface in general can be regarded as another evolutionary playground to tune photoresponses in phytochromes.

Similarly, the PHY-tongue composition has been optimized during evolution of phytochromes to favor either the β-hairpin or a partially α-helical conformation (or potentially intermediate conformations between the two extremes) that either inhibit or stimulate light signal transduction to diverse effector domains by specific sets of interactions with the NTS and the GAF domain. Within the context of our constructs addressing PHY-tongue replacements, the presence of the PHY-tongue of *Ts*PadC in interaction with the *Is*PadC chromophore-binding region does not impair signal transduction; however, it reduces the dynamic range. This property is even more pronounced after the additional incorporation of the *Ts*PadC NTS where almost no stimulation of DGC activity can be observed. Moreover, the rate constants of thermal reversion to Pr changed drastically upon addition of a different NTS element, which supports the role of the NTS element in influencing the stability of the PHY-tongue conformation. Strikingly, the replacement of only the NTS and PHY-tongue elements in *Is*PadC did not impair light signal transduction but obviously strongly affected the stability of the α-helical conformation of the PHY-tongue element defining the Pfr state. This renders the system poorly active in the dark but favors switching between the coiled-coil registers upon illumination and thereby allows strong photoactivation of enzymatic activity. The low dark-state activity and high light stimulation result in a dynamic range of roughly 800-fold; in combination with the fast thermal inactivation, this could be an ideal system for optogenetic applications targeted via c-di-GMP signaling.

In summary, phytochrome sensors have sparked much excitement over the last years. Their capacity to use biliverdin as cofactor, which is present in most mammalian tissues, as a photoreactive trigger for being activated in the near-IR window of light renders bacteriophytochromes very attractive targets for fluorescence imaging (51, 52) and optogenetic tool development (50, 53–55). However, the translation of mechanistic ideas of photoactivation from one photoreceptor to another, even from closely related systems like those described in this study, prove challenging, because of the fine-tuned conformational dynamics of each structural element constituting the photosensory module and the coevolution of residues in different structural elements of each system. Therefore the functional involvement of various phytochrome elements, especially the NTS or NTE elements of bacteriophytochromes or plant phytochromes, respectively, and the PHY-tongue, needs to be understood in the light of their tightly coupled conformational dynamics.

## Experimental procedures

### Protein preparation, expression, and purification

Using pETM-11–based PadC plasmids (15) as templates, we generated chimeric variants by swapping structural domains between two prototypical bacteriophytochrome-linked diguanylyl cyclases, *Is*PadC and *Ts*PadC, using a PCR-based Gibson cloning approach (primers are listed in Table S1).

PadC variants were expressed and purified as described in detail previously (15). To sum up, His_6_-tagged holoproteins were expressed in *E. coli* BL21 (DE3) containing the previously generated pT7-ho1 helper plasmid (15) required for the coexpression of the heme oxygenase (HO-1) involved in bilin chromophore production. The transformed cells were then cultivated at 37 °C in LB medium to an *A*_600_ of 0.5 before being cooled to 16 °C and supplemented with 10 mg liter^−1^ δ-aminolevulinic acid prior to induction with 0.25 mm isopropyl-β-d-thiogalactopyranoside for ∼15 h. The cells were harvested and lysed by combined treatment with lysozyme (100 μg ml^−1^) and sonication (2 × 5 min, 100 W, continuous mode; Labsomic L, Sartorius), before separation of the soluble fraction by ultracentrifugation (206,000 × *g*).

Soluble proteins were affinity-purified on a Ni^2+^-Sepharose matrix (Ni^2+^-Sepharose 6 Fast Flow; GE Healthcare). After a washing step (lysis buffer containing 30 mm imidazole), proteins were eluted (lysis buffer containing 250 mm imidazole) and dialyzed overnight at 4 °C in dialysis buffer (50 mm Hepes, pH 7, 500 mm NaCl, 2 mm MgCl_2_, 1 mm dithioerythritol) in the presence of histidine-tagged tobacco etch virus (TEV) protease (∼1:16 TEV/substrate). Cleaved histidine tag and TEV protease were removed from the purified protein by reloading the dialyzed proteins onto the Ni^2+^ column and collecting the flow through. Collected proteins were subjected to an additional step of size-exclusion chromatography on a 16/60 Superdex 200 prep grade column (GE Healthcare) before being concentrated (Amicon Ultra-15; Merck Millipore) in storage buffer (10 mm Hepes, pH 7, 500 mm NaCl, 2 mm MgCl_2_), aliquoted, flash-frozen in liquid nitrogen, and conserved at −80 °C until needed.

Prior to steady-state kinetics characterization of the protein variants, proteins were subject to an additional size-exclusion chromatography run on a 10/300 Superdex 200 increase analytical grade column (GE Healthcare) to exchange the protein into HPLC buffer (50 mm Hepes, pH 7, 500 mm NaCl, 50 mm MgCl_2_) and remove potential protein aggregates caused by sample thawing.

### Cell-based diguanylyl cyclase assay

Diguanylyl cyclase activity was screened following our previously described protocol (15). pETM-11 PadC-based plasmids were transformed in *E. coli* BL21 (DE3) containing the pT7-ho1 plasmid and grown at 30 °C to *A*_600_ 0.5 in YESCA medium (casamino acids 10 mg ml^−1^ and yeast extract 1.5 mg ml^−1^) supplemented with MgSO_4_ (0.05 mg ml^−1^), FeSO_4_ (0.5 μg ml^−1^), kanamycin (30 μg ml^−1^), and chloramphenicol (34 μg ml^−1^). After 4 h of induction at 16 °C with 0.25 mm isopropyl-β-d-thiogalactopyranoside in the presence of 10 mg liter^−1^ δ-aminolevulinic acid, the induced cultures were concentrated to an *A*_600_ of 10, and 5 μl of the concentrated cultures were spotted on YESCA agar plates containing 30 μg ml^−1^ kanamycin, 34 μg ml^−1^ chloramphenicol, and 0.01 mg ml^−1^ Congo Red and incubated at 20 °C for 16 h in the dark or under constant red light illumination (630 nm, 75 μW cm^−2^). As negative control we used a pETM-11 AppA construct that does not show any DGC activity.

### Spectrophotometry of protein samples

UV-visible absorption spectra were acquired with a CCD-based Specord S300 Vis spectrophotometer (Analytic Jena) using protein samples diluted in storage buffer and equilibrated at 20 °C. Dark-adapted Pr-state absorption spectra were recorded under nonactinic light conditions by minimizing the contact time with measuring light using a neutral density filter corresponding to an optical density of 2.0 (Thorlabs) between the light source and the sample cuvette. Pfr-enriched spectra were recorded under constant red light irradiation (660 nm, 45 mW cm^−2^; Thorlabs) in the presence of the same neutral density filter for the measuring light.

Pr-state recovery kinetics were followed at the maximal wavelength of the Pr-state Q-band after 1-min red light illumination (660 nm, 45 mW cm^−2^; Thorlabs) using a Specord 200 Plus spectrophotometer (Analytic Jena) with 10-ms integration time sampled every 5 s. Even the lowest integration time possible on this device influences the recovery kinetics of constructs with extremely slow thermal recoveries. To enable a qualitative comparison of all constructs, we extracted the theoretical end point of the recoveries from a dark-adapted spectrum acquired prior to the illumination of the samples. This end point was then included as a fixed value to obtain estimations of the very slow recovering components. The values extracted via this process should not be overinterpreted quantitatively but serve to highlight the complex recovery kinetics and the markedly different characteristics of the individual chimeras. Similarly, only few data points are available for fitting the first contributions of very fast recovering constructs; however, to facilitate a comparison of all constructs, a sampling interval of 5 s was an acceptable compromise.

### Spectroscopic characterization of biliverdin isomerization

UV-visible absorption spectra of the denatured biliverdin bound proteins were measured as described previously (36). The concentrated protein sample was kept in the dark or irradiated 1 min with saturating red light (660 nm, 45 mW cm^−2^; Thorlabs), before diluting 1:10 in quenching buffer (0.1% TCA in methanol) to a final concentration of 1–2 μm. The absorption spectra of the denatured Pr or Pfr states were then directly recorded. The *ZZZssa* isomer has a maximum of absorption at 708 nm, and the *ZZEssa* isomer has its absorption maximum at 624 nm.

### c-di-GMP production kinetics

To record the conversion of GTP to c-di-GMP for all the variants, we adapted a protocol (15) for HPLC. Briefly, purified protein samples were mixed with GTP at various concentrations in reaction buffer (50 mm Hepes, pH 7, 500 mm NaCl, and 50 mm MgCl_2_) at 20 °C under nonactinic light for the dark-state measurement or under constant red light illumination (660 nm, 45 mW cm^−2^; Thorlabs) following a 1-min preillumination of the sample for the light state measurements. Samples were then thermally inactivated by 1 min of incubation at 95 °C. After separating the substrate and products from denatured protein by centrifugation, the nucleotides were separated by a linear 7-min gradient from 2 to 20% MeOH using a reversed phase HPLC column (SunFire C18 4.6 × 100; Waters) equilibrated in 10 mm triethylammonium formate (pH 6.0). For protein variants isolated in a partially activated state and featuring a slow thermal recovery, a prolonged incubation of the sample under far-red light (730 nm ± 20 nm; Thorlabs LED) coupled to a far-red light bandpass filter (750 nm; Thorlabs) at room temperature and complete darkness was performed to fully populate the Pr state of the sample prior to the dark state measurements. Denaturation in methanol with 0.1% TCA of part of the recovered sample was done prior to dark state measurement to confirm the presence of more than 95% of the chromophore population in *ZZZssa* isomer. All kinetic data were normalized to the concentration of the dimeric protein, and all samples were corrected for the amount of c-di-GMP formed during the inactivation step.

## Author contributions

G. G. and A. W. data curation; G. G. formal analysis; G. G. and A. W. supervision; G. G. and U. V. investigation; G. G. visualization; G. G., U. V., and A. W. methodology; G. G. writing-original draft; A. W. conceptualization; A. W. resources; A. W. funding acquisition; A. W. project administration; A. W. writing-review and editing.

## Supplementary Material

Supporting Information

## Footnotes

2 The abbreviations used are:

PSM: photosensory module
PAS: period/ARNT/single-minded
NTS: N-terminal segment
GAF: cGMP phosphodiesterase/adenylyl cyclase/FhlA
PHY: phytochrome-specific
DGC: diguanylyl cyclase
NTE: N-terminal extension
TEV: tobacco etch virus.
