# Supplementary material for: Influence of the N-terminal segment and the PHY-tongue element on light-regulation in bacteriophytochromes
Source: J Biol Chem. 2019 Jan 25;294(12):4498–510. doi: 10.1074/jbc.RA118.007260 (PMC6433076; doi:10.1074/jbc.RA118.007260)
Supplement: Supporting Information [file supp_294_12_4498__index.html]

Influence of the N-terminal segment and the PHY-tongue element on light-regulation in bacteriophytochromes — Photoactivation of full-length bacteriophytochromes — Influence of the N-terminal segment and the PHY-tongue element on light-regulation in bacteriophytochromes — Photoactivation of full-length bacteriophytochromes — Supporting Information 

# Influence of the N-terminal segment and the PHY-tongue element on light-regulation in bacteriophytochromes

## Supporting Information

- Supporting Information (to be published online) - Figures S1 to S4 Table S1
