# Supplementary material for: Influence of the N-terminal segment and the PHY-tongue element on light-regulation in bacteriophytochromes
Source: J Biol Chem. 2019 Jan 25;294(12):4498–510. doi: 10.1074/jbc.RA118.007260 (PMC6433076; doi:10.1074/jbc.RA118.007260)
Supplement: Supporting Information [file supp_RA118.007260_142731_1_supp_272721_plqbh4.pdf]

Supplementary Information for

Influence of the N-terminal segment and the PHY-tongue element on light-regulation in bacteriophytochromes.

Geoffrey Gourinchas[1], Ursula Vide[1], and Andreas Winkler[1,2*].

From the [1]Institute of Biochemistry, Graz University of Technology, Petersgasse 12/II, 8010 Graz, Austria; [2]BioTechMed-Graz, 8010 Graz, Austria.

*Correspondence: Andreas.Winkler@TUGraz.at

This PDF file includes:

Figures S1 to S4

Table S1

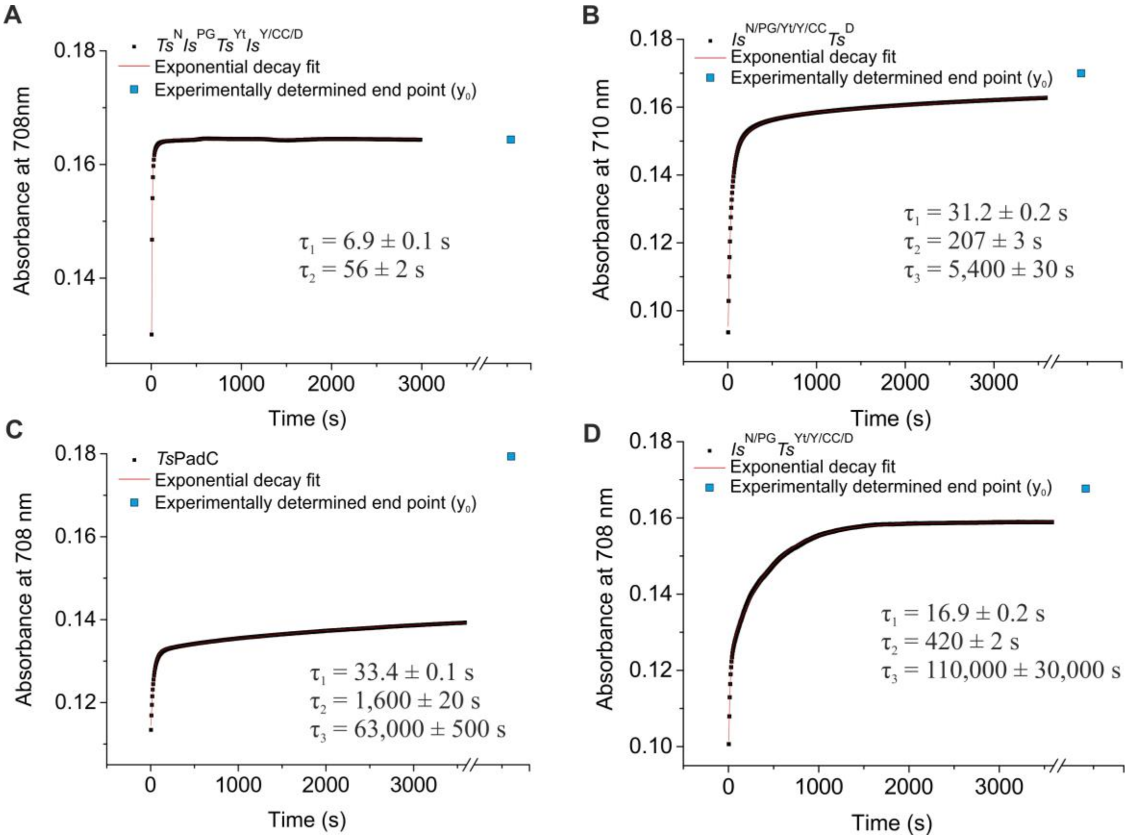

Figure S1. Exponential fitting of representative thermal recoveries. Panels **(A)** to **(D)** represent dark state recovery kinetics of representative constructs featuring very fast to very slow thermal recoveries. Time traces correspond to the maximum absorbance of the Q-band in Pr after 1 min red light illumination for a protein sample of 2 μM equilibrated at 20 °C. Absorbance values were recorded every 5 sec and fitted using the sum of two or three exponential decay functions with the additional restraint of a fixed end point (y_0) extracted from the previously measured dark state spectrum (More details in the *Experimental Procedures*).

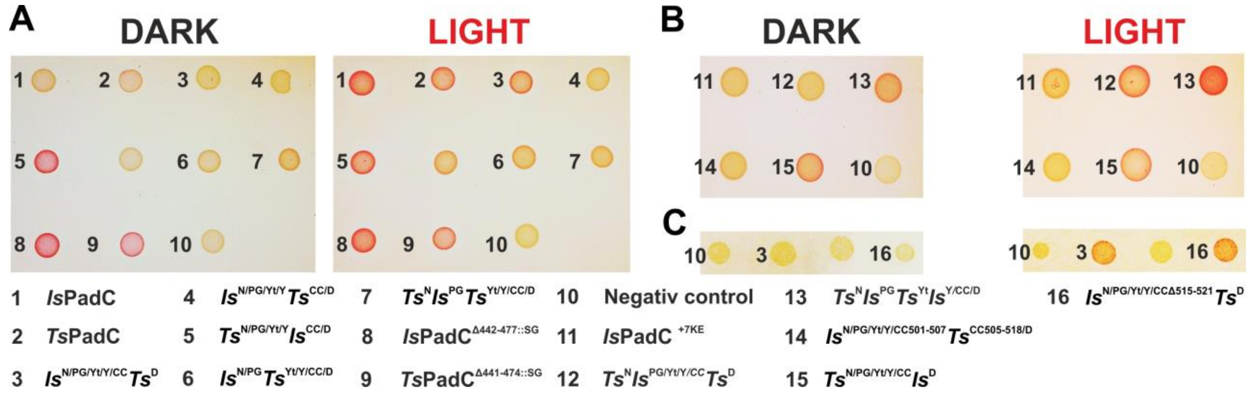

Figure S2. ***In vivo*** **screening of DGC activity.** From (**A**) to (**C**) are six different screening plates from where individual colonies have been extracted for Fig. 1 to facilitate the comparison of constructs. Colonies without number associated are constructs which are not discussed further in this study.

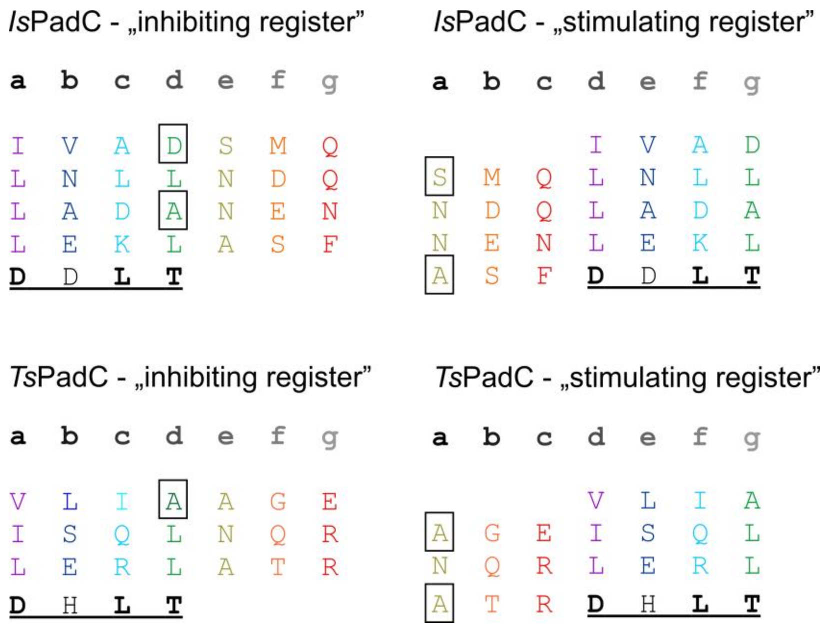

Figure S3. *Is*PadC and *Ts*PadC coiled-coil linker registers. Schematic representation of the inhibiting and stimulating coiled-coil register of *Is*PadC and *Ts*PadC. Destabilizing residues are boxed.

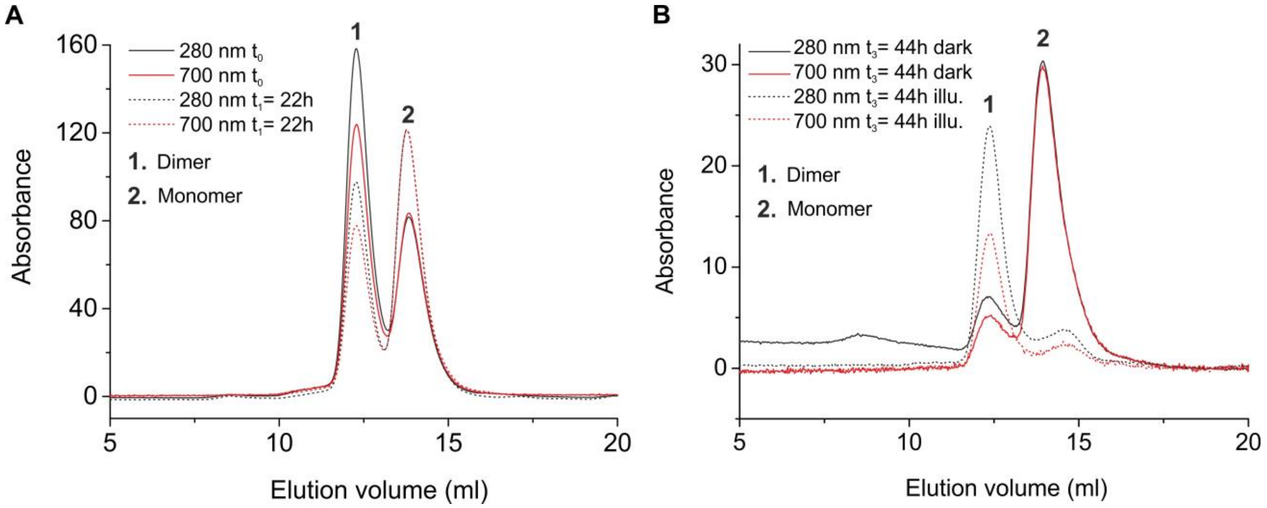

Figure S4. *Ts*$^{\mathbf{N/PG/Yt/Y}}$*Is*$^{\mathbf{CC/D}}$ monomerization upon dark state recovery. (A) Gel filtration analysis of the time dependent monomerization of *Ts*$^{N/PG/Yt/Y}$*Is*$^{CC/D}$ during Pr-state recovery using a Superdex200 10/300. **(B)** After 44h in the dark a sample of *Ts*$^{N/PG/Yt/Y}$*Is*$^{CC/D}$ was illuminated for 1 min with red light and analyzed again via gel filtration under constant illumination. Redimerization of the full-length protein supports the reversibility of the monomer/dimer equilibrium of this construct.

Table S1. Overview of oligonucleotides and buffers. Related to figure 1. (A) Oligonucleotides used in this study. **(B)** Buffers used for purification and storage of the different *Is*PadC variants.

Panel **A** - oligonucleotides

Construct	Oligonucleotide (5‘-3‘)	Template
$Ts^{\mathrm{N/PG/Yt/Y}}Is^{\mathrm{CC/D}}$	fw: atctggcaagcgatctggccattgtggcagatagcatg<br>rv: tgctcgagtgcggccgcttactggctacaaacctgattac	*Is*PadC
	fw: taagcggccgcactcgag<br>rv: ggccagatcgcttgccag	*Ts*PadC
$Is^{\mathrm{N/PG/Yt/Y/CC}}Ts^{\mathrm{D}}$	fw: taagcggccgcactcgag<br>rv: atcaaagctggccagtttttcc	*Is*PadC
	fw: tggaaaaactggccagctttgatcatctgaccggtctg<br>rv: tgctcgagtgcggccgcttaatctgcgctaacttcaac	*Ts*PadC
$Is^{\mathrm{N/PG/Yt/Y}}Ts^{\mathrm{CC/D}}$	fw: taagcggccgcactcgag<br>rv: cagcagatcacgtgcaatatcac	*Is*PadC
	fw: atattgcacgtgatctgctggttctgattgcagccggt<br>rv: tgctcgagtgcggccgcttaatctgcgctaacttcaacg	*Ts*PadC
$Is^{\mathrm{N/PG/Yt/Y/CC501\text{-}507}}Ts^{\mathrm{CC505\text{-}518/D}}$	fw: attgtggcagatagcatgcagattagccagctgaatcagc<br>rv: ctgcatgctatctgccacaatcagcagatcacgtgc	$Is^{\mathrm{N/PG/Yt/Y}}Ts^{\mathrm{CC/D}}$
$Is^{\mathrm{N/PG}}Ts^{\mathrm{Yt/Y/CC/D}}$	fw: taagcggccgcactcgag<br>rv: atgaatcagccacagacgttct	*Is*PadC
	fw: cgtctgtggctgattcatgcacgtgatgatgcagttctgat<br>rv: tgctcgagtgcggccgcttaatctgcgctaacttcaacg	*Ts*PadC
$Ts^{\mathrm{N}}Is^{\mathrm{PG}}Ts^{\mathrm{Yt/Y/CC/D}}$	fw: tggttgcagcactggaagcatgtgaacgt gaaccgattcatattccgaatgcaattcagc<br>rv: cttccagtgctgcaaccagttccggttcatccat ggcgccctgaaaataaagattctcagtagtg	$Is^{\mathrm{N/PG}}Ts^{\mathrm{Yt/Y/CC/D}}$
$IsPadC^{PSM}$	fw: gcacgtgatctgctg taagcggccgcactcg<br>rv: cagcagatcacgtgc aatatcacgtgctgcatacagctg	*Is*PadC
$Is^{\mathrm{N/PG/Yt/Y/CC\Delta 515\text{-}521}}Ts^{\mathrm{D}}$	fw: aatgatcagctggaaaaactggccagctttgatcatctgaccggtctgtg<br>rv: ctgatcattcagcagattcagc	$Is^{\mathrm{N/PG/Yt/Y/CC}}Ts^{\mathrm{D}}$
$Ts^{\mathrm{N/PG/Yt/Y/CC}}Is^{\mathrm{D}}$	fw: tggaacgtctggcaacccgtgatgatctgaccggtatc<br>rv: tgctcgagtgcggccgcttactggctacaaacctgattac	*Is*PadC
	fw:taagcggccgcactcgag<br>rv: acgggttgccagacgttc	*Ts*PadC
$Ts^{\mathrm{N}}Is^{\mathrm{PG/Yt/Y/CC}}Ts^{\mathrm{D}}$	fw: tggttgcagcactggaagcatgtgaacgtgaaccgattcatattccgaatgcaattcagc<br>rv: cttccagtgctgcaaccagttccggttcatccatggcgccctgaaaataaagattctcagtagtg	$Is^{\mathrm{N/PG/Yt/Y/CC}}Ts^{\mathrm{D}}$
$Ts^{\mathrm{N}}Is^{\mathrm{PG}}Ts^{\mathrm{Yt}}Is^{\mathrm{Y/CC/D}}$	fw: agcggtaaaagccagc<br>rv: ctgtgcaacacgaaacag	$Ts^{\mathrm{N}}Is^{\mathrm{PG/Yt/Y/CC}}Ts^{\mathrm{D}}$
	fw: ctgtttcgtgttgcacaggcagaaacccgtatttgggcag<br>rv: gctggcttttaccgctaacggtttctttccagcttgcaaag	*Ts*PadC

Panel **B** – buffer systems

Use	Buffer composition
Storage buffer	10 mM HEPES pH 7, 0.5 M NaCl, 2 mM $MgCl_2$
Lysis buffer	50 mM HEPES pH 7, 0.5 M NaCl, 2 mM $MgCl_2$, 10 mM imidazole
Dialysis buffer	50 mM HEPES pH 7, 0.5 M NaCl, 2 mM $MgCl_2$, 1 mM DTE
HPLC buffer	50 mM HEPES pH 7, 0.5 M NaCl, 50 mM $MgCl_2$
